# Supplementary material for: Nuclear-import receptors remodel the dilute phase to suppress phase transitions of RNA-binding proteins with prion-like domains
Source: bioRxiv. 2025 Nov 15:2025.11.14.688546. Preprint. [Version 1] doi: 10.1101/2025.11.14.688546 (PMC12642399; doi:10.1101/2025.11.14.688546)
Supplement: 1 [file NIHPP2025.11.14.688546V1-supplement-1.pdf]

bioRxiv preprint doi: https://doi.org/10.1101/2025.11.14.688546; this version posted November 15, 2025. The copyright holder for this preprint (which was not certified by peer review) is the author/funder, who has granted bioRxiv a license to display the preprint in perpetuity. It is made available under aCC-BY 4.0 International license.

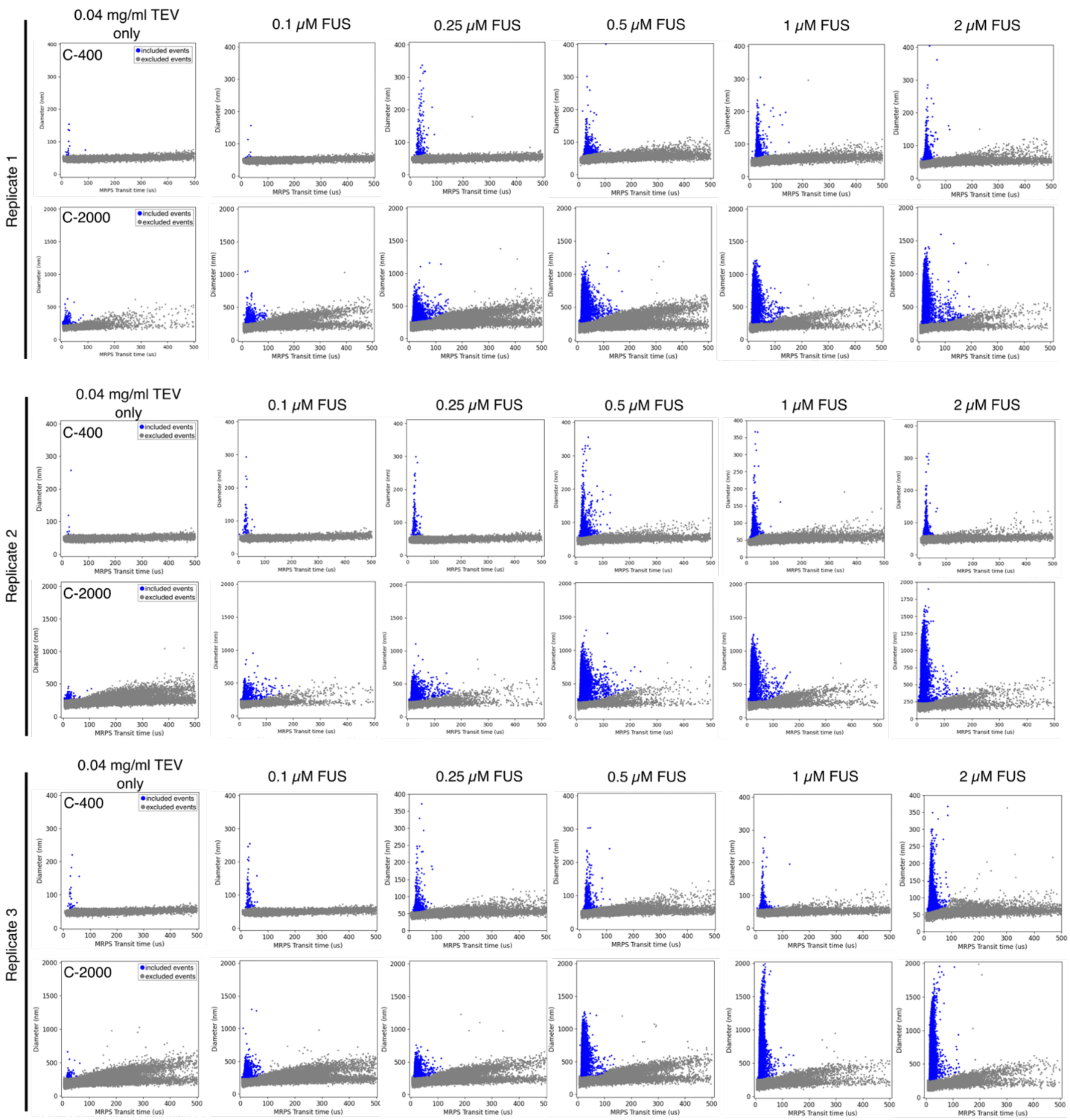

Supplementary Fig. 1: Microfluidic Resistive Pulse Sensing (MRPS) scatter plots of samples at increasing FUS concentrations. Three independent replicates of each condition were measured using two types of cartridges with different nano constriction widths (C-400 and C-2000). 0.04 mg/ml TEV protease was measured as control. Scatter plots show the measured particle diameter as a function of the MRPS transit time. Each blue dot represents a single event, corresponding to a single particle crossing the nano constriction. These scatter plots were used to extract size distributions shown in Fig. 2b. Gray dots represent the noise floor of the instrument and were excluded from the analysis.

bioRxiv preprint doi: https://doi.org/10.1101/2025.11.14.688546; this version posted November 15, 2025. The copyright holder for this preprint (which was not certified by peer review) is the author/funder, who has granted bioRxiv a license to display the preprint in perpetuity. It is made available under aCC-BY 4.0 International license.

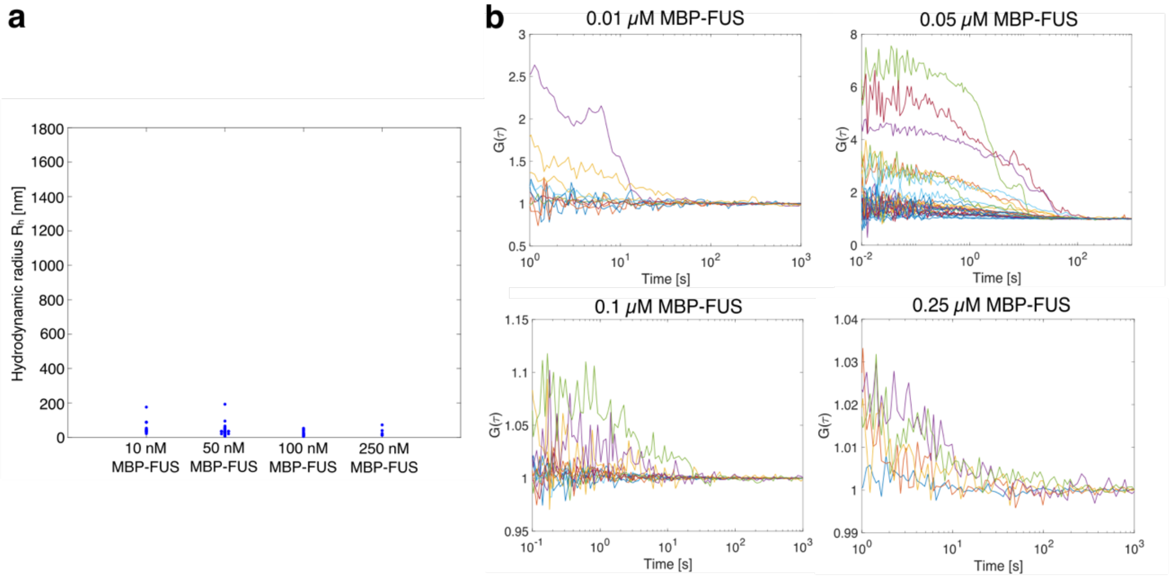

Supplementary Fig. 2: Fluorescence Correlation Spectroscopy (FCS) of MBP-FUS in absence of TEV protease. a, Without TEV protease, MBP-FUS particle sizes remain largely below 100 nm. **b,** FCS autocorrelation functions of samples in a.

bioRxiv preprint doi: https://doi.org/10.1101/2025.11.14.688546; this version posted November 15, 2025. The copyright holder for this preprint (which was not certified by peer review) is the author/funder, who has granted bioRxiv a license to display the preprint in perpetuity. It is made available under aCC-BY 4.0 International license.

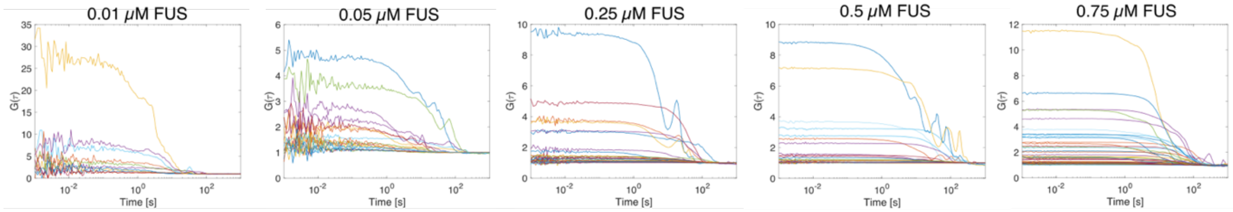

Supplementary Fig. 3: FCS autocorrelation functions of samples at increasing FUS concentrations. Autocorrelation functions were extracted from fluorescence intensity fluctuations over 10 s. A single-component model was fitted to determine characteristic diffusion times τ_D and particle hydrodynamic radii R_h. Particle sizes are showed in Fig. 2c.

bioRxiv preprint doi: https://doi.org/10.1101/2025.11.14.688546; this version posted November 15, 2025. The copyright holder for this preprint (which was not certified by peer review) is the author/funder, who has granted bioRxiv a license to display the preprint in perpetuity. It is made available under aCC-BY 4.0 International license.

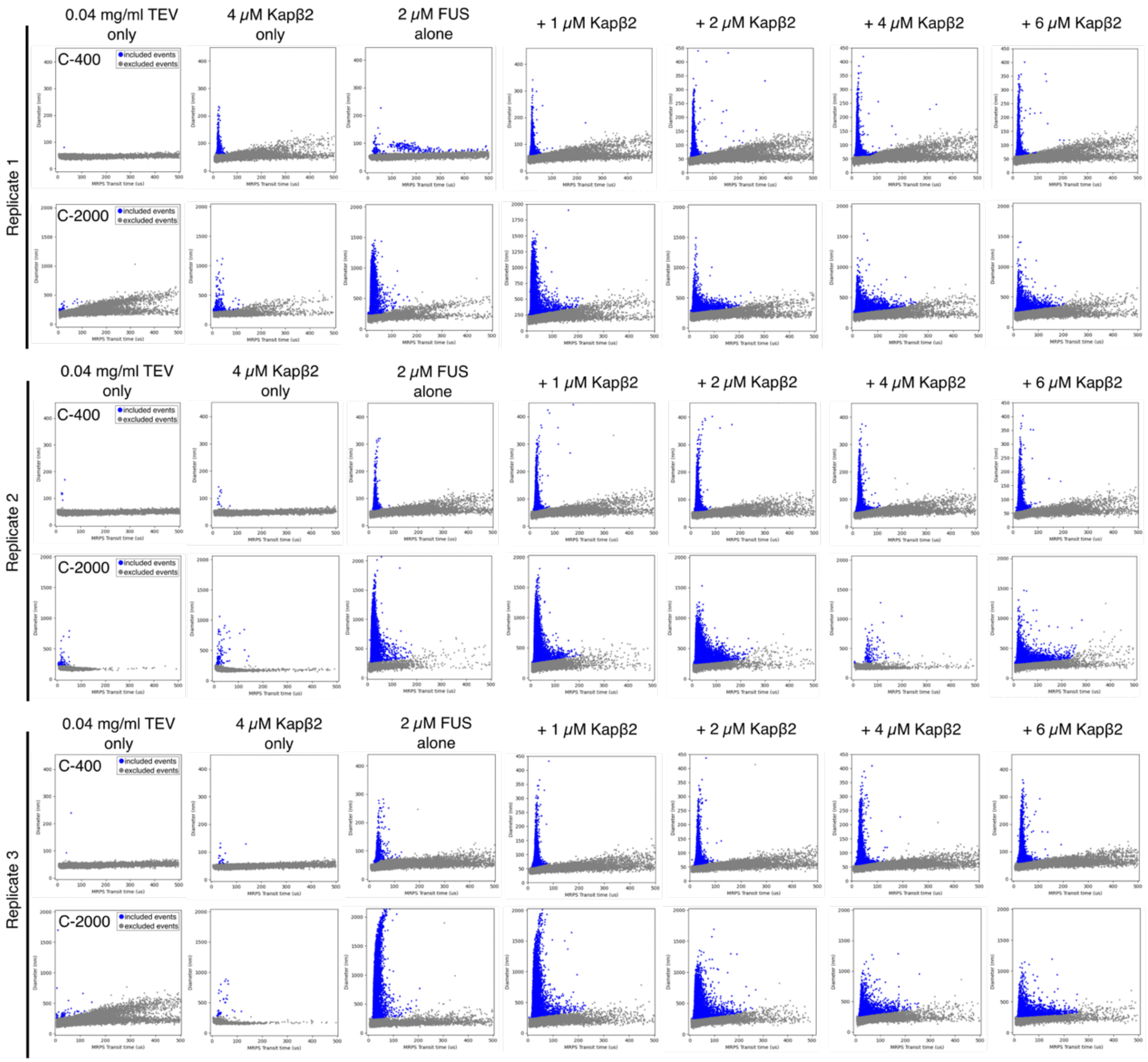

Supplementary Fig. 4: Microfluidic Resistive Pulse Sensing (MRPS) size scatter plots of samples at 2 µM FUS in absence and presence of increasing Kapβ2 concentrations. 0.04 mg/ml TEV protease and 4 µM Kapβ2 alone were analyzed as control samples. Each condition was measured as three independent replicates with two types of cartridges (C-400 and C-2000). Scatter plots show the measured particle diameter as a function of the MRPS transit time. Blue dots represent single assemblies which were used to extract size distributions depicted in Fig. 2d. Gray dots correspond to the noise floor of the instrument and were excluded from the analysis.

bioRxiv preprint doi: https://doi.org/10.1101/2025.11.14.688546; this version posted November 15, 2025. The copyright holder for this preprint (which was not certified by peer review) is the author/funder, who has granted bioRxiv a license to display the preprint in perpetuity. It is made available under aCC-BY 4.0 International license.

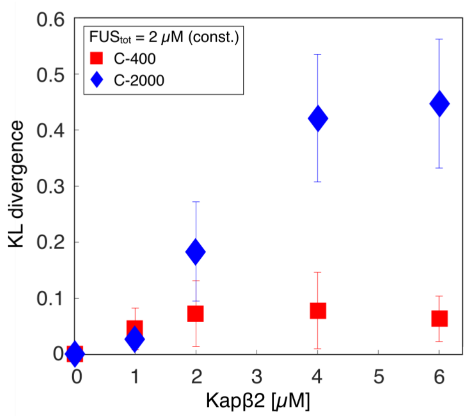

Supplementary Fig. 5: Kullback-Leibler (KL) divergence of size distributions of FUS in presence of Kapβ2 with respect to FUS alone. The KL divergence increases with increasing Kapβ2 concentrations, plateauing at ≥ 4 µM Kapβ2. Mean KL divergence was obtained from size distributions measured as triplicates using C-400 and C-2000 cartridges. Error bars represent the standard deviation.

bioRxiv preprint doi: https://doi.org/10.1101/2025.11.14.688546; this version posted November 15, 2025. The copyright holder for this preprint (which was not certified by peer review) is the author/funder, who has granted bioRxiv a license to display the preprint in perpetuity. It is made available under aCC-BY 4.0 International license.

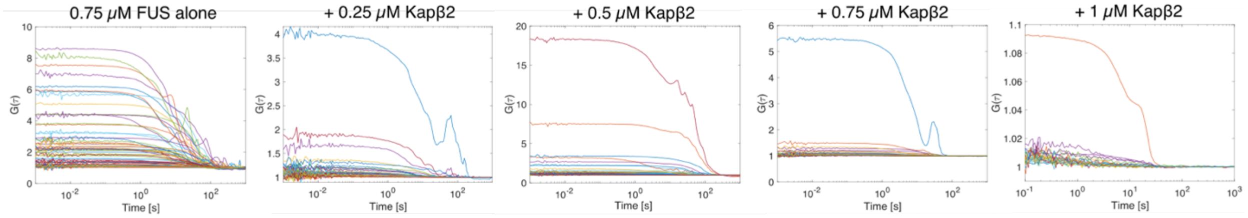

Supplementary Fig. 6: FCS autocorrelation functions of FUS samples in absence and presence of increasing Kapβ2 concentrations. Autocorrelation functions were extracted from fluorescence intensity fluctuations over 10 seconds. A single-component model was used to extract characteristic diffusion times τ_D to compute particle hydrodynamic radii R_h which are shown in Fig. 2f.

bioRxiv preprint doi: https://doi.org/10.1101/2025.11.14.688546; this version posted November 15, 2025. The copyright holder for this preprint (which was not certified by peer review) is the author/funder, who has granted bioRxiv a license to display the preprint in perpetuity. It is made available under aCC-BY 4.0 International license.

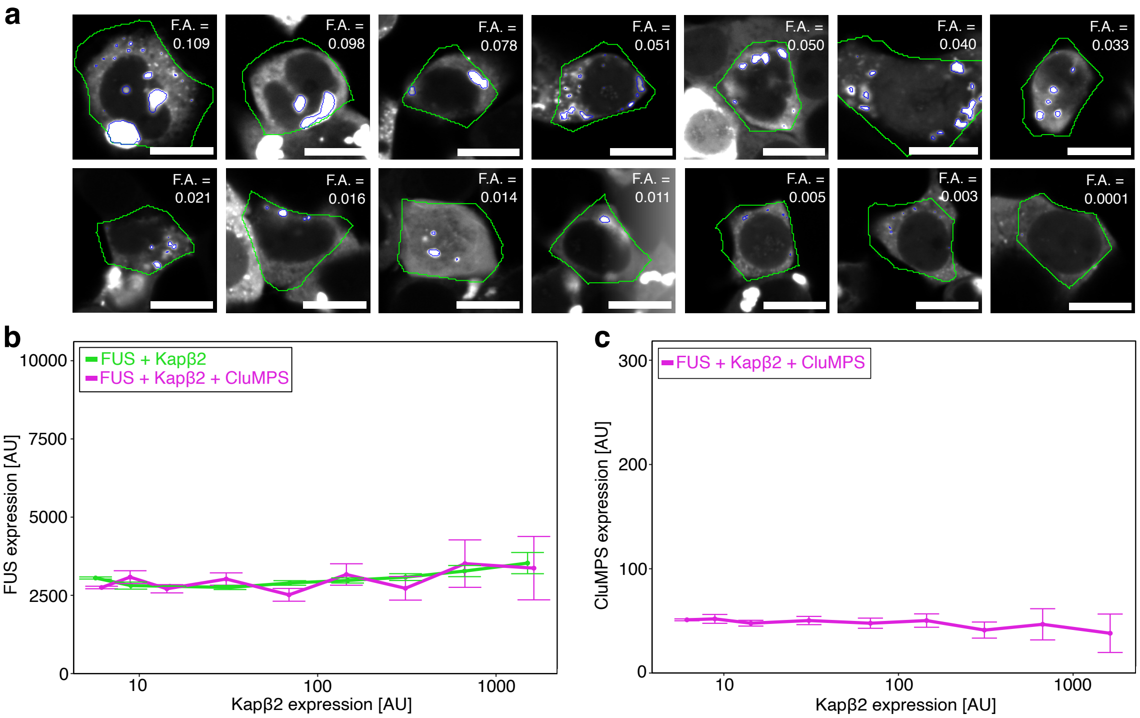

Supplementary Fig. 7: Expression levels of FUS and the CluMPS reporter across increasing Kapβ2 concentrations. a, Representative images of cells exhibiting increasing extent of FUS assembly, as quantified by extracting the fraction of condensate area / cell area (F.A.). Scale bars, 20 µm. **b,** Effect of increasing expression levels of Kapβ2 on FUS condensation is not driven by FUS expression levels. **c,** Effect of increasing expression levels of Kapβ2 on FUS condensation is not driven by expression levels of the CluMPS reporter.

bioRxiv preprint doi: https://doi.org/10.1101/2025.11.14.688546; this version posted November 15, 2025. The copyright holder for this preprint (which was not certified by peer review) is the author/funder, who has granted bioRxiv a license to display the preprint in perpetuity. It is made available under aCC-BY 4.0 International license.

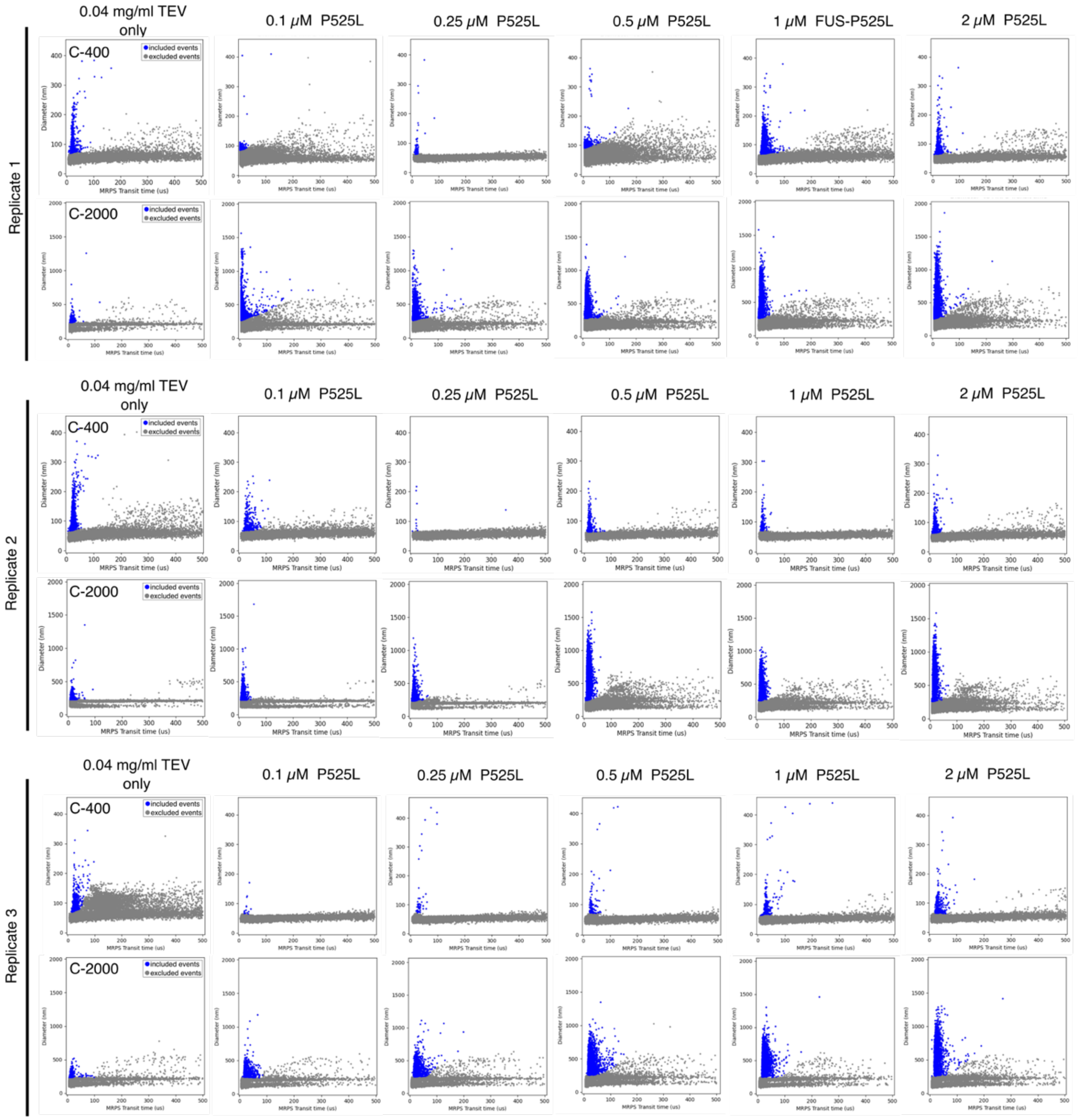

Supplementary Fig. 8: Raw MRPS size scatter plots of samples at increasing FUS^{P525L} concentrations. Scatter plots depict the diameters of single assemblies as a function of the MRPS transit time. Data correspond to size distributions in Fig. 5d. 0.04 mg/ml TEV protease served as control sample. Each blue dot is the diameter of a single particle, gray dots represent the noise floor of the instrument and were excluded. All samples were measured using two types of cartridges with different nano constriction widths (C-400 and C-2000) and replicated three times.

bioRxiv preprint doi: https://doi.org/10.1101/2025.11.14.688546; this version posted November 15, 2025. The copyright holder for this preprint (which was not certified by peer review) is the author/funder, who has granted bioRxiv a license to display the preprint in perpetuity. It is made available under aCC-BY 4.0 International license.

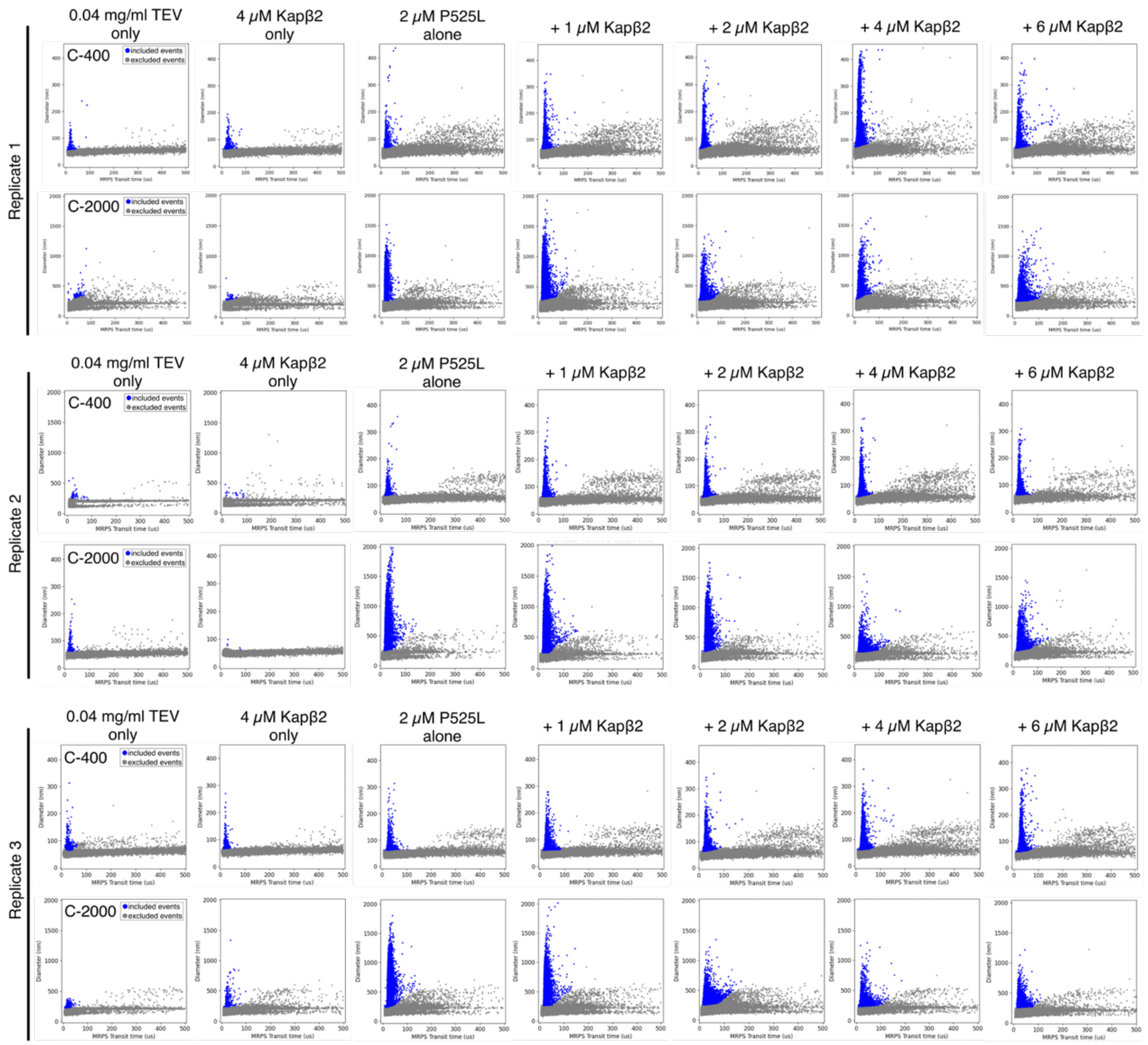

Supplementary Fig. 9: Raw MRPS scatter plots of FUS^{P525L} samples in absence and presence of increasing concentrations of Kapβ2. Plots show three replicates of the particle diameter as a function of the MRPS transit time. Each sample was measured using cartridges with a narrower (C-400) and a wider (C-2000) nano constrictions. 0.04 mg/ml TEV protease and 4 µM Kapβ2 alone were analyzed as control samples. Each blue dot represents a single particle detected. Gray dots correspond to the noise floor of the instrument and were not counted as events. Size distributions extracted from this data are shown in Fig. 5f.

bioRxiv preprint doi: https://doi.org/10.1101/2025.11.14.688546; this version posted November 15, 2025. The copyright holder for this preprint (which was not certified by peer review) is the author/funder, who has granted bioRxiv a license to display the preprint in perpetuity. It is made available under aCC-BY 4.0 International license.

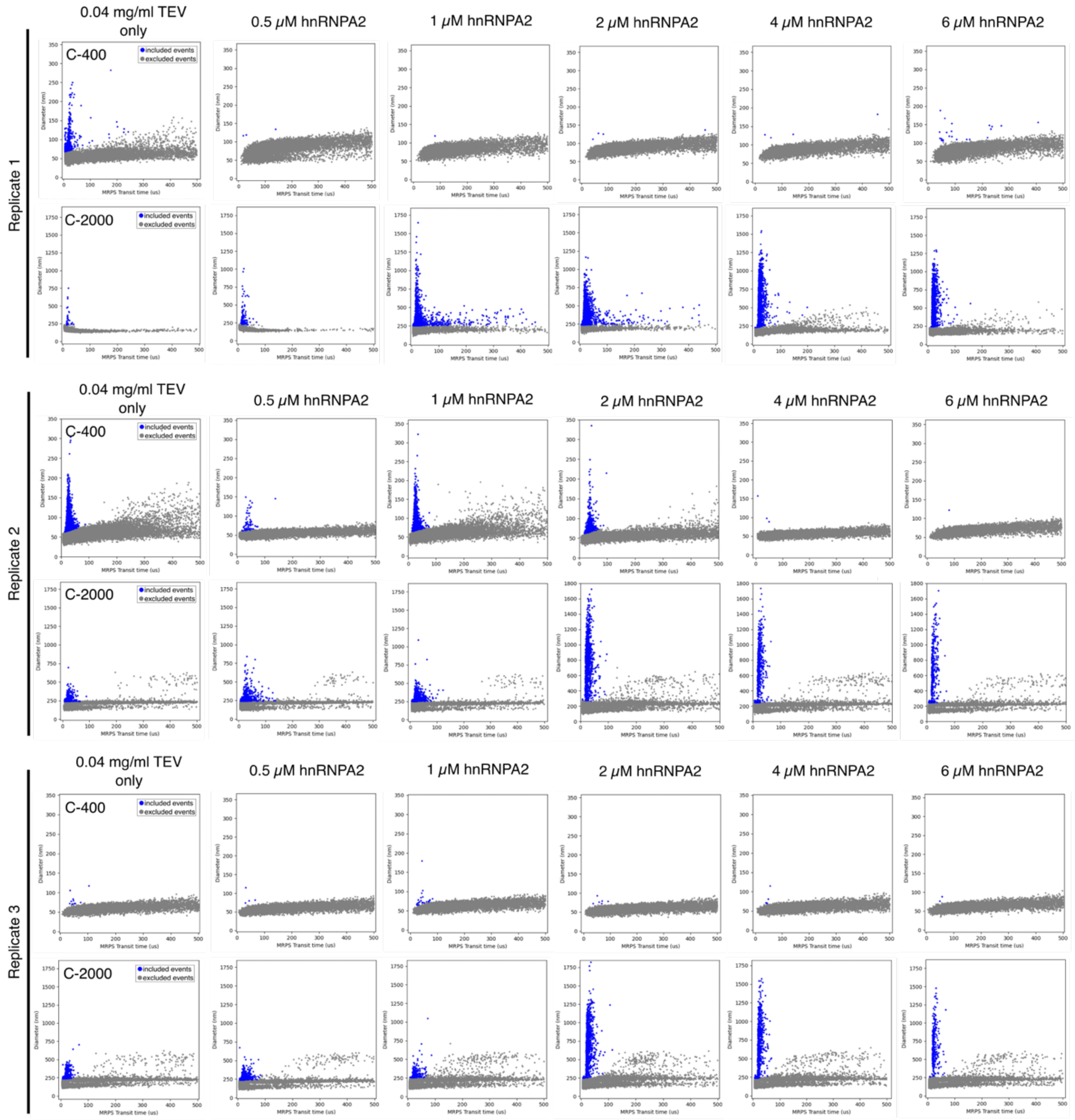

Supplementary Fig. 10: Raw MRPS scatter plots of samples at increasing hnRNPA2 concentrations. Plots show the particle diameter dependent on the MRPS transit time. Each blue dot is a single particle crossing the nano constriction. All samples were measured in triplicates using two different cartridges (C-400 and C-2000) to capture a larger range of particle sizes. 0.04 mg/ml TEV protease was measured as control. Gray dots represent the noise floor. Scatter plots were used to extract particle size distributions depicted in Fig. 6b.

bioRxiv preprint doi: https://doi.org/10.1101/2025.11.14.688546; this version posted November 15, 2025. The copyright holder for this preprint (which was not certified by peer review) is the author/funder, who has granted bioRxiv a license to display the preprint in perpetuity. It is made available under aCC-BY 4.0 International license.

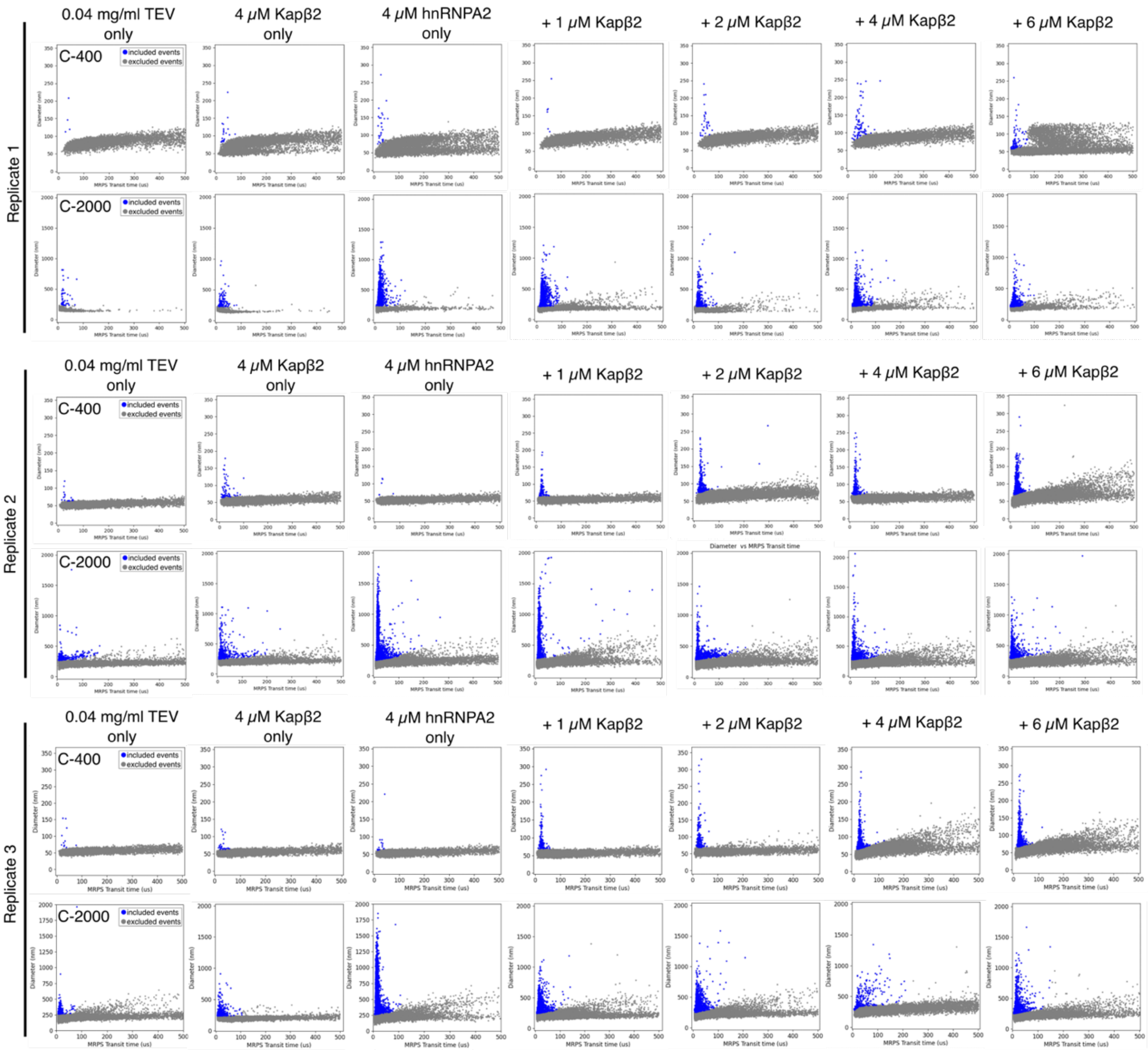

Supplementary Fig. 11: Raw MRPS scatter plots of hnRNPA2 in absence and presence of increasing Kapβ2 concentrations. Measurements were carried out as triplicates. Two different microfluidic cartridges (C-400 and C-2000) were used to cover a large range of particle sizes. 0.04 mg/ml TEV protease and 4 μM Kapβ2 alone were used as controls. Gray dots are the noise floor of the instrument. Blue dots represent single assemblies of distinct sizes which were used to extract size distributions depicted in Fig. 6d.

bioRxiv preprint doi: https://doi.org/10.1101/2025.11.14.688546; this version posted November 15, 2025. The copyright holder for this preprint (which was not certified by peer review) is the author/funder, who has granted bioRxiv a license to display the preprint in perpetuity. It is made available under aCC-BY 4.0 International license.

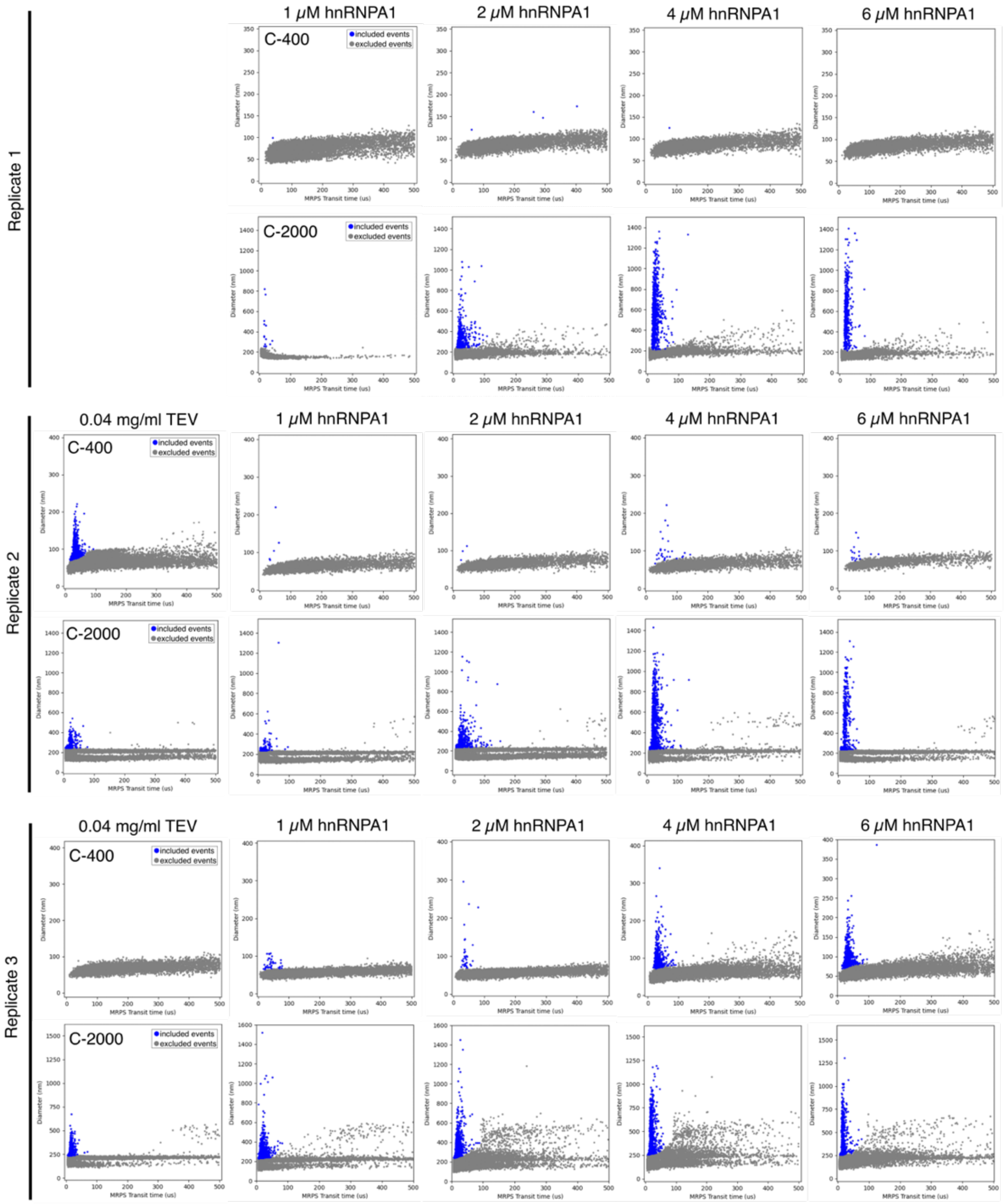

Supplementary Fig. 12: Raw MRPS scatter plots of samples at increasing hnRNPA1 concentrations. Plots depict the particle diameter in nm versus the MRPS transit time in s. Each sample was measured in three replicates. 0.04 mg/ml TEV protease served as control sample. Each blue dot represents a single event of a particle crossing the nano constriction in the respective cartridge (C-400 and C-2000). Size distributions corresponding to this data are shown in Extended Data Fig. 4b.

bioRxiv preprint doi: https://doi.org/10.1101/2025.11.14.688546; this version posted November 15, 2025. The copyright holder for this preprint (which was not certified by peer review) is the author/funder, who has granted bioRxiv a license to display the preprint in perpetuity. It is made available under aCC-BY 4.0 International license.

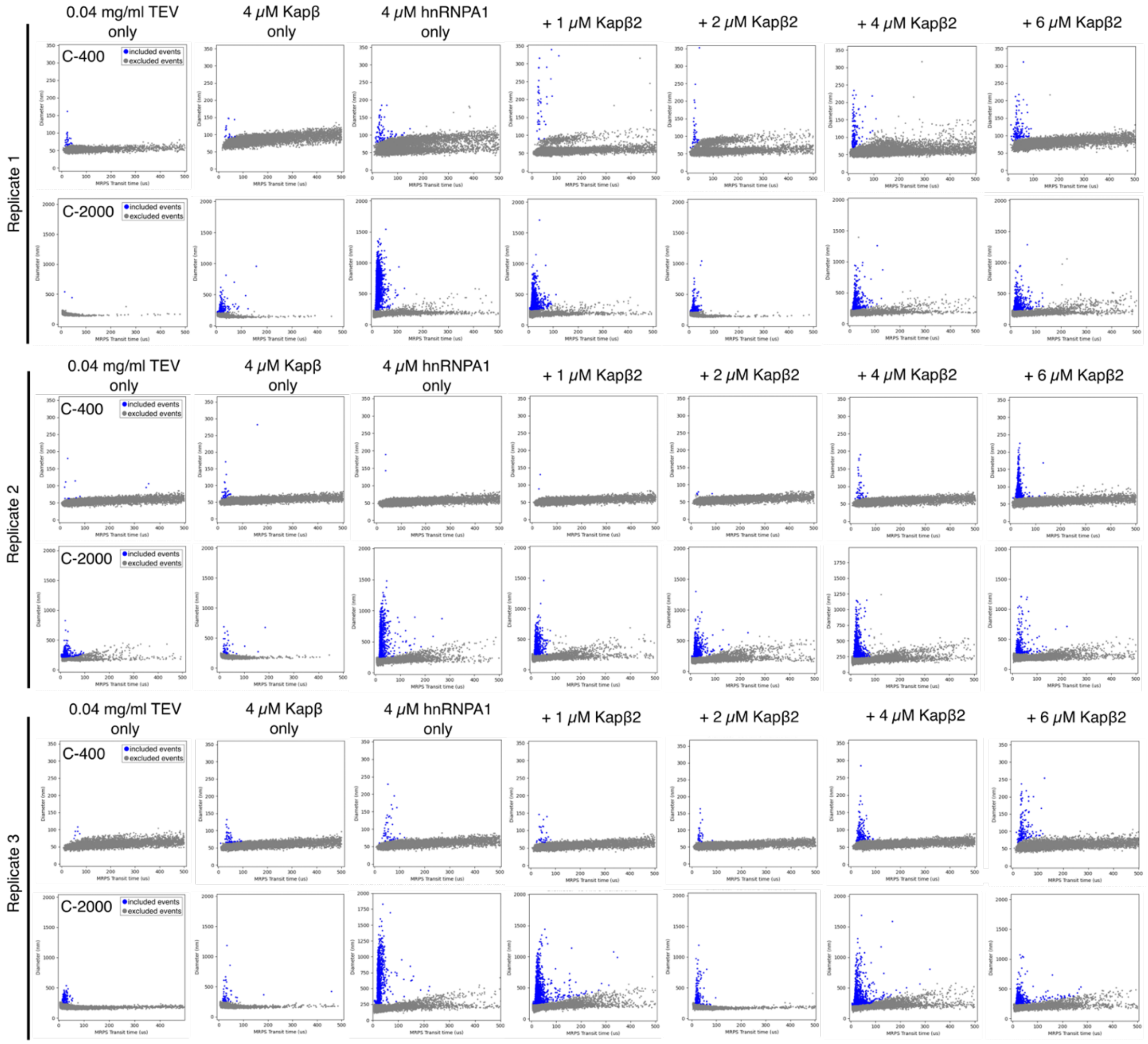

Supplementary Fig. 13: Raw MRPS scatter plots of hnRNPA1 in absence and presence of increasing Kapβ2 concentrations. Scatter plots show the particle diameter versus the MRPS transit time. Three independent replicates of each sample were measure. 0.04 mg/ml TEV protease and 4 μM Kapβ2 alone were analyzed as controls. All samples were measured using cartridges of two different sizes (C-400 and C-2000). Blue dots represent single assemblies, gray dots are the noise of the instrument and were excluded from the analysis. Corresponding size distributions are shown in Extended Data Fig. 4d.

bioRxiv preprint doi: https://doi.org/10.1101/2025.11.14.688546; this version posted November 15, 2025. The copyright holder for this preprint (which was not certified by peer review) is the author/funder, who has granted bioRxiv a license to display the preprint in perpetuity. It is made available under aCC-BY 4.0 International license.

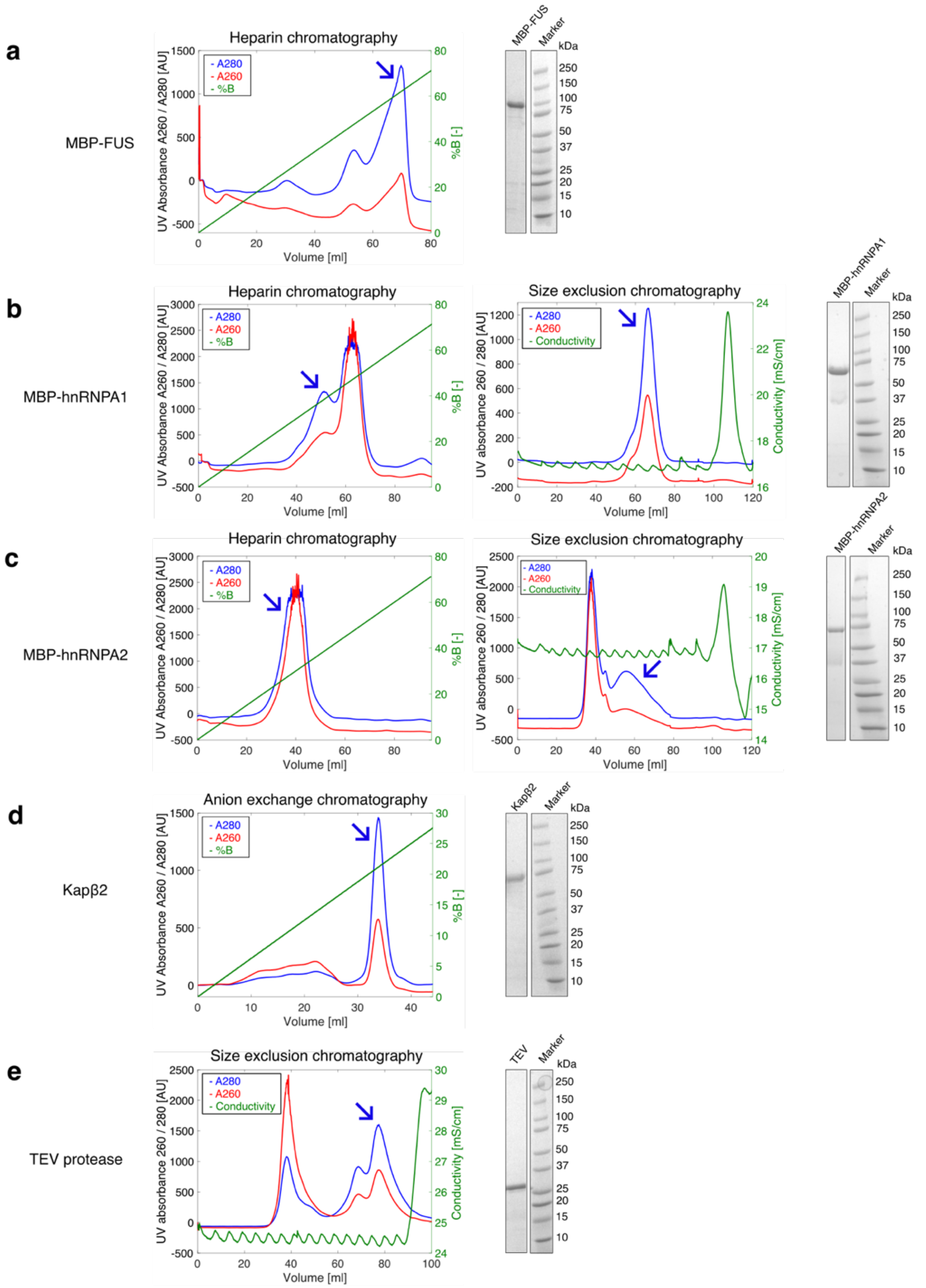

Supplementary Fig. 14: Purification of all proteins used in this study. a, Purification of MBP-FUS via Amylose-based affinity chromatography and Heparin chromatography (The right panel shows a representative SDS-PAGE demonstrating protein purity. **b,** MBP-hnRNPA1 was purified using amylose-based affinity chromatography, Heparin chromatography to remove bound RNA, and SEC. SDS-PAGE was used to confirm purity of final product. **c,** MBP-hnRNPA2 was purified using the same procedure as MBP-hnRNPA2. **d,** Kapβ2 was purified using Nickel-NTA affinity chromatography followed by anion exchange chromatography. SDS-PAGE confirms protein

bioRxiv preprint doi: https://doi.org/10.1101/2025.11.14.688546; this version posted November 15, 2025. The copyright holder for this preprint (which was not certified by peer review) is the author/funder, who has granted bioRxiv a license to display the preprint in perpetuity. It is made available under aCC-BY 4.0 International license.

purity. **e,** TEV protease was purified via Ni-NTA affinity chromatography and SEC. The final protein product was pure, as shown via SDS-PAGE. In all panels, arrows indicate the peak containing the protein-of-interest for further purification or to be stored.
